# Readiness of health facilities to deliver family planning services and associated factors in urban east-central Uganda

**DOI:** 10.1186/s12978-025-02026-w

**Published:** 2025-05-15

**Authors:** Jacquellyn Nambi Ssanyu, Rornald Muhumuza Kananura, Leif Eriksson, Peter Waiswa, Mats Målqvist, Joan Nakayaga Kalyango

**Affiliations:** 1 Department of Health Policy Planning and Management, School of Public Health, Makerere University, Kampala, Uganda; 2 Department of Women’s and Children’s Health, Centre for Health and Sustainability, Uppsala University, Uppsala, Sweden; 3 African Population and Health Research Center, Nairobi, Kenya; 4 Department of Global Public Health, Karolinska Institute, Stockholm, Sweden; 5 Busoga Health Forum, Jinja, Uganda; 6 Department of Pharmacy, School of Health Sciences, Makerere University, Kampala, Uganda

**Keywords:** Health facility readiness, Family Planning, Urban, Uganda, Access barriers

## Abstract

**Background:**

Health facility readiness is essential for realizing voluntary, rights-based family planning. However, many countries, including rapidly urbanizing Uganda, face challenges in ensuring their health facilities are sufficiently equipped to meet the growing demand for these services. This study assessed readiness and associated factors across public, private-not-for-profit (PNFP), and private-for-profit (PFP) health facilities in urban east-central Uganda to guide strategies for improving service delivery.

**Methods:**

The study used secondary data from a cross-sectional study done in Jinja City and Iganga Municipality, including a health facility assessment and health worker survey. Readiness was measured using the Service Availability and Readiness Assessment methodology, and health worker knowledge and biases were assessed through the Situation Analysis approach. Sample weights adjusted for facility and health worker representation, and linear regression examined associations between readiness scores and various factors.

**Results:**

Among 152 health facilities, 94.2% offered family planning services, with an average readiness score of 46.7% (standard deviation ± 17.0). Short-acting methods had high availability (99.0%), while long-acting reversible contraceptives (34.2%) and permanent options (8.9%) were less available, compounded by prevalent stock-outs. Additionally, staff refresher training was inadequate, particularly in PFP facilities (50.4%), and health worker knowledge, confidence and willingness to provide some methods, particularly long-acting options and natural family planning counselling, were low. Notably, out of 261 health workers, 97.7% imposed at least one restriction to service access based on either age, parity, marital status, or spousal consent, more pronounced in PNFP facilities. Readiness was significantly associated with facility level (health centre level II facilities: β = -9.42, *p* = 0.036; drug shops: β = -11.00, *p* = 0.022), external supervision (β = 9.04, *p* = 0.009), holding administrative meetings (β = 9.72, *p* = 0.017), and imposing marital status (β = -9.42, *p* = 0.017) and spousal consent access barriers (β = 6.24, *p* = 0.023).

**Conclusions:**

This study found sub-optimal facility readiness, highlighting the need to strengthen governance of services across both public and private sectors, implement comprehensive training for health workers in both sectors, and align policies to ensure equitable access to a full range of services for all clients.

**Supplementary Information:**

The online version contains supplementary material available at 10.1186/s12978-025-02026-w.

## Abstract (plain language summary)

Health facility readiness is crucial for ensuring that family planning services are available and accessible to everyone. However, many countries, including Uganda, still struggle to ensure their facilities are equipped to meet the growing demand for these services, especially in urban areas. This study looked at the readiness of public, private-not-for-profit (PNFP), and private-for-profit (PFP) health facilities in urban east-central Uganda. The study used data from 152 health facilities in Jinja City and Iganga Municipality. It assessed how prepared these facilities were to offer family planning services and also looked at health workers’ knowledge and biases. The results showed that while most facilities provided family planning services, the overall readiness score was low (46.7%). Short-acting methods were widely available, but long-acting methods were less available and stock-outs were common. Additionally, health workers’ training was insufficient, especially in PFP facilities, and they lacked confidence in providing some methods, such as long-term options and natural family planning counselling. Moreover, the study found that many health workers (97.7%) imposed restrictions on access to services, based on age, number of children, marital status, and spousal consent, with these being more common in PNFP facilities. Factors including facility level, supervision from external authorities, holding administrative meetings, and imposing access barriers were significantly associated with facility readiness. The study concludes that improving facility readiness requires better governance of family planning services in both public and private sectors, improving health worker training, and updating policies to ensure all clients can access a full range of family planning options.

**Supplementary Information:**

The online version contains supplementary material available at 10.1186/s12978-025-02026-w.

## Background

Increasing access to family planning services is crucial for improving maternal and child health outcomes and enhancing reproductive autonomy [1, 2]. Achieving this requires ensuring health facilities are ready to deliver the intended standard of care, a core component of family planning program quality [3, 4]. Facility readiness encompasses adequate infrastructure, reliable contraceptive supplies, functional information systems, as well as having adequately trained staff [5].

Several global initiatives have been undertaken to expand access to family planning services [6], aligning with Sustainable Development Goal 3.7 [7]. However, many low- and middle-income countries continue to struggle in ensuring that their health facilities are sufficiently equipped to meet the growing demand [8, 9]. In Uganda, for instance, while family planning services are widely available, with one study indicating a 92% availability [9], facility readiness to provide these services is low [10, 11], with frequent stock-outs and gaps in provider training undermining service delivery [12–14]. These challenges hinder the realisation of a voluntary, rights-based approach to family planning, essential for individuals to exercise their reproductive rights and make informed, autonomous choices [15].

In Uganda’s urban areas, unplanned rapid population growth strains the already overwhelmed public health systems, leaving them unable to meet the needs of urban dwellers. Consequently, Private-For-Profit (PFP) facilities, often located closer to communities, become the main source of family planning services for many urban residents [16]. However, the quality and consistency of services provided by these private facilities are questionable due to poor regulation and supervision [17]. Moreover, the profit-driven nature of PFP facilities may result in an incomplete family planning method mix, as they may prioritise high-demand and high-profit methods. Research also shows disparities in the distribution and quality of family planning services in Uganda’s urban areas, with formal settlements generally having better-equipped facilities, with more trained staff, compared to informal ones [18]. This leads to unequal access to services and can further widen health inequalities between wealthier and poorer urban populations.

The gaps in facility readiness manifest as poor client experiences such as inadequate counselling [14], increased costs of care [12], and inadequate response to concerns about contraceptive-related adverse events [19, 20], which in turn reduce family planning uptake and continued use. These issues likely contribute to the 15.1% unmet need for family planning in Uganda’s urban areas [21]. To address this, strengthening both public and private healthcare systems is essential for Uganda to effectively implement its Total Market Approach (TMA)—a sustainable resource mobilization framework that leverages the full spectrum of public, private, and donor providers to expand access to family planning services [22]. This approach will ensure equitable access to affordable, high-quality family planning services for all, regardless of ability to pay.

However, facility readiness, particularly in private-sector facilities, remains under-studied in Uganda. National and regional surveys typically sample from facilities that report to the Health Management Information System, a limited subset of the total private healthcare landscape. This exclusion leaves gaps in the understanding of service provision, especially in the private sector where many clients seek care. Comprehensive assessments of facility readiness, across both public and private sectors, are crucial for identifying service delivery weaknesses and guiding policy and programmatic interventions to improve family planning service availability, accessibility, and quality. To this end, this study aimed to assess facility readiness across public, Private-Not-For-Profit (PNFP), and PFP health facilities in urban areas of east-central Uganda, and to identify the factors associated with readiness.

## Methods

### Study design and setting

Data used in this study were collected in February and March 2022 as part of a larger project, the Urban Thrive Project [23], which implemented system-strengthening interventions to increase the uptake of voluntary family planning in urban east-central Uganda. These secondary data were derived from a cross-sectional study that included a health facility assessment and health worker survey done as part of the project’s baseline evaluation.

The study was conducted in Jinja City and Iganga Municipality, two growing urban areas in the Busoga sub-region of east-central Uganda. Busoga has a high total fertility rate of 5.7, compared to the national average of 5.2, with only 36.2% of married women using modern contraception [21]. Additionally, 21.8% of women have an unmet need for family planning, one of the highest rates in the country [21]. Jinja, located in Jinja District, is Uganda’s second-largest city and is located 80 km from Kampala, the country’s capital. It has a population of 307,414 people [24]. Iganga Municipality, with a population of 55,263 [25], is Iganga District’s administrative and commercial centre and lies about 45 km northeast of Jinja City [26].

**Fig. 1:**
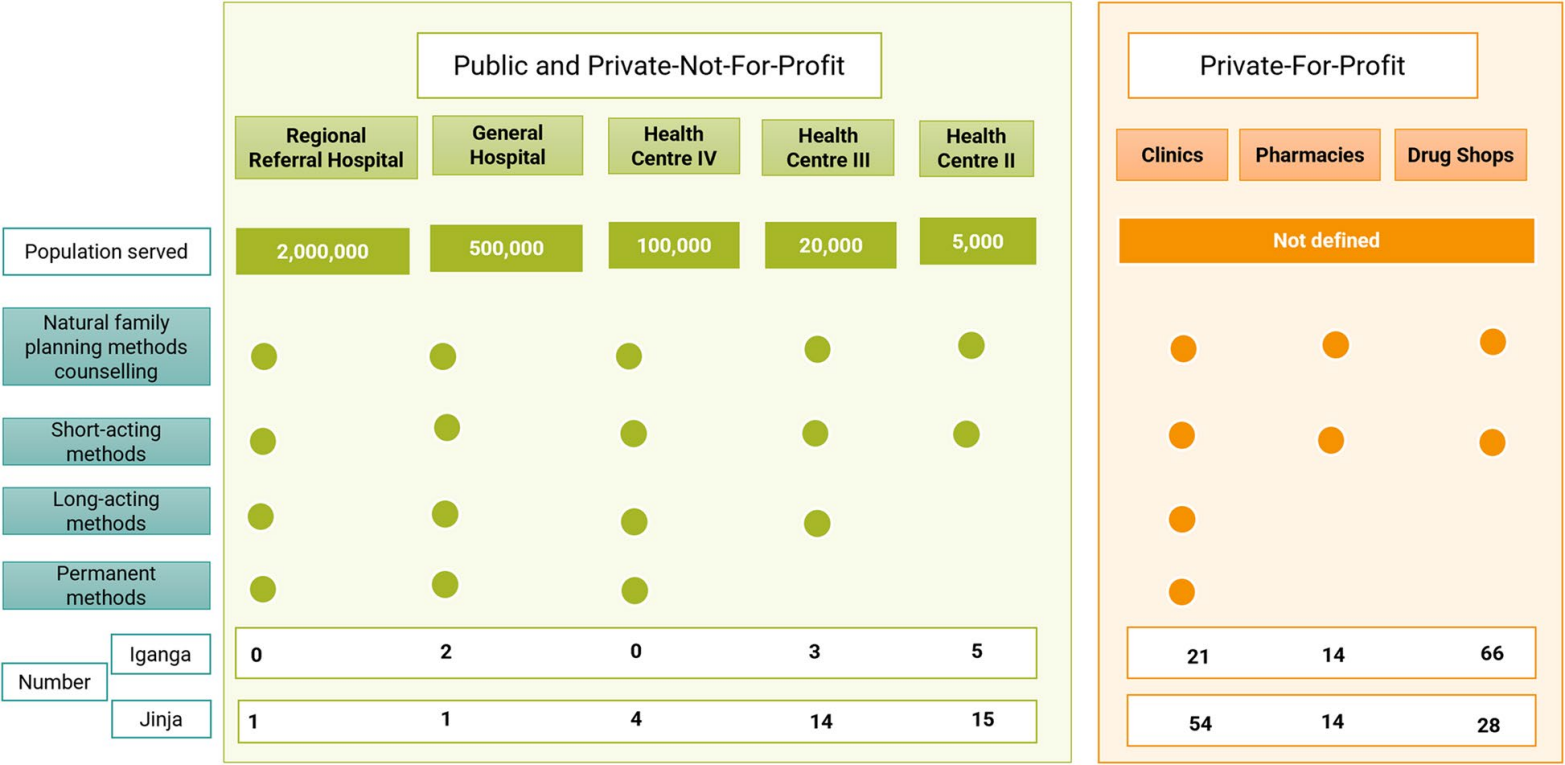
Family planning services provided at each level and the distribution of health facilities across levels

Family planning services in Uganda are provided through three types of facilities: public facilities, where services are free; PNFP facilities (both faith-based and those run by Non-Governmental Organizations (NGOs)), which offer subsidized services; and PFP facilities, including private hospitals, clinics, pharmacies, and drug shops, where services are fully priced. Public and PNFP facilities operate within a seven-level, referral-based structure, ranging from community-level services, including Community Health Workers (CHWs), to national referral hospitals. This structure is organized based on service complexity and the population served [27]. Several NGOs complement these facility-based services through community outreaches and demand-generation activities. A facility listing done prior to the study showed that Iganga Municipality had 111 health facilities, with the majority (66) being private drug shops, while Jinja had 131 facilities, with private clinics comprising the largest group (54) (Fig. 1).

Modern family planning methods available in Uganda include short-acting, long-acting reversible, and permanent options. Short-acting methods include non-prescription options such as barrier methods (male and female condoms) and fertility awareness-based methods like Lactational Amenorrhea Method (LAM), Standard Days Method (SDM), and moon beads. Prescription-only methods in this category include oral contraceptive pills and injectables (Depot Medroxyprogesterone Acetate (DMPA) in both intra-muscular (IM) and the recently introduced self-administered subcutaneous (SC) forms). Long-acting reversible methods, available by prescription, include implants and intra-uterine devices (IUDs). Permanent options, such as tubal ligation and vasectomy, are also prescription-only [28].

Method availability varies by facility level. Among public health facilities, Health centre (HC) II facilities offer the fewest options, as they are not equipped to provide long-acting or permanent options (Fig. 1). HCIIIs provide all methods except permanent methods, while HCIV and higher facilities offer the full range of methods. Private clinics’ offerings depend on capacity and demand. Private pharmacies, under the National Drug Policy and Authority Act, are authorized to dispense both non-prescription and prescription family planning methods with a licensed medical practitioner’s recommendation [29], though many prescription-only methods are also available unofficially over-the-counter. Drug shops are typically licensed to provide only non-prescription products. However, due to research and advocacy from implementing partner organizations, they now stock and administer oral contraceptive pills, DMPA-IM, and DMPA-SC without a prescription [22, 30, 31]. CHWs, on the other hand, can provide and administer all short-term methods, as well as offer counselling for natural family planning methods.

### Participant selection and study size

The study included public, PNFP, and PFP facilities in Jinja City and Iganga Municipality. PFP facilities comprised licensed and unlicensed clinics, pharmacies, and drug shops, excluding herbal clinics, veterinary pharmacies, veterinary drug shops, and domiciliaries. Additionally, health workers in the selected facilities were included. Eligible health workers were those 18 years or older, employed for at least three months, and involved in providing any Sexual Reproductive Health (SRH) services.

The sample size for health facilities was calculated using Kish [32]’s formula for cross-sectional studies, assuming 50% of health facilities were ready to provide family planning services, a 95% confidence level and 5% precision, giving 385 facilities. However, since the total number of health facilities at both sites was lower (111 in Iganga and 131 in Jinja), a finite population adjustment was applied, along with a 10% non-response rate, expected especially in PFP facilities, resulting in sample sizes of 75 facilities for Iganga and 90 for Jinja. All public, PNFP, and higher-level PFP facilities (hospitals) were included, while drug shops, pharmacies, and clinics were selected using systematic random sampling from the list of mapped facilities. After restricting the sample to only those facilities within the bounds of Jinja City and Iganga Municipality, data from only 152 facilities were included in the study.

For health workers, Kish [32]’s formula, with a 50% readiness assumption and a 95% confidence level, yielded a sample of 385. Probability proportional to size sampling was used to determine the number of health workers to select from facilities at different levels. In facilities with multiple eligible health workers, convenience sampling was used to choose the individuals for interview. For this study, only data from health workers in eligible facilities were included.

### Data collection procedures

Data collection from health facilities involved interviewing family planning focal persons and facility managers, verifying responses on-site for questions that required confirmation, and recording the data using a checklist. Health worker data were gathered using an interviewer-administered questionnaire in English. Both tools had been pre-tested in two facilities, which were excluded from the analysis. All data were collected electronically with Kobocollect [33] installed on tablets by four trained research assistants with nursing backgrounds, under the supervision of the lead author.

### Variables

The outcome variable was facility readiness to provide family planning services, assessed using the Service Availability and Readiness Assessment (SARA) methodology, a systematic survey that uses tracer indicators of service availability and readiness to assess health facility service delivery [34]. Readiness was measured using family planning-specific tracer items, categorized into three domains: 1) Staff and guidelines; 2) Equipment; and 3) Medicines and commodities [34]. Tracer indicators and their definitions are detailed in Additional File 1. Readiness was assessed only for facilities that provided family planning services, with each facility evaluated based on the services it was mandated to offer at its level of care at the time of data collection.

Independent variables included site (Jinja or Iganga); facility level (categorized as higher-level facilities, HCIII, HCII, private pharmacies, private drug shops, and private clinics); ownership type (public, PNFP, or PFP); and location (slum or non-slum area). Additionally, we assessed governance-related factors, including whether facilities received external supervision for family planning services and held administrative meetings with documented records.

Health workers’ biases in providing different family planning methods (pills, condoms, IUDs, injectables, or sterilization) were also assessed using questions adopted from the Situation Analysis tool [35]. A minimum age barrier was considered present if providers set a limit of 10 years (adolescent age) or older to accessing any of the five methods. A parity barrier was implied if health workers required any number of prior births to access any of the methods. A marital status barrier was indicated by a refusal to provide any of the methods to unmarried women. Finally, a spousal consent barrier was identified if providers required a husband’s consent to access any of the methods [35]. The proportion of health workers imposing each barrier was aggregated at the facility level.

### Statistical methods

Data collected on Kobocollect were exported to Microsoft Excel for cleaning and subsequently imported into Stata (version 14.2) for analysis. Sample weights were calculated for each health facility by dividing the number of facilities at each level of care by the total number of facilities at that level in the area. Similarly, sample weights for health workers were determined by dividing the number of health workers interviewed at each facility by the total number of health workers in relevant SRH-providing cadres, and then multiplying this quotient by the facility weight. These weights were applied to all relevant analyses. Descriptive statistics for health facilities and workers were presented as frequencies with weighted proportions for categorical variables and means with standard deviations (SDs) for continuous variables.

To generate readiness scores, facilities were scored 1 if each tracer item was available and 0 if it was not. The total score for each facility was calculated, divided by the maximum possible score for that facility’s level of care, and multiplied by 100 to determine the facility’s percentage readiness score. Linear regression was then used to assess the association between various factors and readiness through bivariate analysis. Variables with *p-*values less than 0.2 were considered for inclusion in the multivariable model. Stepwise elimination was then applied to refine the model by evaluating R-squared (*R*^*2*^) values to assess the contribution of each variable and model fit, based on the Akaike Information Criterion. Collinearity was assessed between ownership and facility level, as certain facility types (clinics, pharmacies, and drug shops) were only found in PFP facilities, while higher-level facilities were predominantly public or PNFP. To address potential collinearity, the model included only the level of care, which provided the better model fit. Statistical significance was defined as a *p-*value < 0.05.

## Results

### Health facility and health worker background characteristics

Out of the 152 health facilities included, 54.3% were from Jinja (Table 1). Most facilities were located in non-slum areas (79.2%), and the majority were PFP facilities (73.9%). According to level of facility, drug shops were the most prevalent, comprising 38.4% of the sample, followed by clinics at 31.5%.

**Table 1:** Characteristics of 152 health facilities included from Jinja City and Iganga Municipality

**Characteristic**	**Sample frequency** (*N* = 152)	**Weighted Percentage**
Site		
Iganga Municipality	76	45.7
Jinja City	76	54.3
Slum location		
No	124	79.2
Yes	28	20.8
Ownership		
Public	32	13.2
Private-Not-For-Profit	19	12.9
Private-For-Profit	101	73.9
Level of facility		
Higher level facilities^a^	8	3.3
Health Centre IIIs	17	7.0
Health Centre IIs	20	8.2
Private Pharmacies	14	11.6
Private Drug Shops	57	38.4
Private Clinics	36	31.5

^a^Regional Referral Hospital (1), General Hospital (both public and private) (3), and Health Centre IV (4)

Health workers were interviewed from each included health facility, except for one clinic in Jinja. Of the 174 health workers interviewed, most (68.3%) were female, and 44.5% were between the ages of 21 and 30 (Table 2). Most participants (67.3%) were either married or cohabiting, and 32.4% identified as Anglican. Nearly half (46.1%) were employed in higher-level health facilities, while only 5.0% worked in pharmacies. Nurses and midwives formed the largest professional group, with enrolled nurses/midwives representing 40.3% and registered nurses accounting for 26.7%. In contrast, only one pharmacist and one pharmacy technician participated, while CHWs constituted 12.1% of the sample. Furthermore, 67.7% of the participants had completed their basic training over four years prior, and nearly half (49.3%) reported working at their current health facility for one to four years. Majority (67.4%) of these health workers worked in facilities in Jinja.

**Table 2:** Health worker characteristics (*N* = 174)

**Characteristic**	**Sample Frequency** (*N* = 174)	**Weighted Percentage**
Age		
21 to 30 years	130	44.5
31 to 40 years	82	32.9
41 to 72 years	62	22.6
Gender		
Female	202	68.3
Male	72	31.7
Marital status		
Never Married	81	27.0
Married/Cohabiting	176	67.3
Separated/Widowed	17	5.7
Denomination		
Pentecostal	68	27.5
Catholic	61	22.0
Moslem	45	15.3
Anglican	88	32.4
Seventh Day Adventist	12	2.8
Level of health facility		
Higher level facility	103	46.1
Health Centre II	40	8.0
Pharmacy	15	5.0
Drug Shop	59	9.4
Clinic	57	31.5
Cadre		
Medical Officer	7	3.6
Clinical Officer	35	14.5
Pharmacist/Pharmacy Technician	2	0.6
Registered Nurse/midwife	49	26.7
Enrolled Nurse/midwife	117	40.3
Nursing Assistant	5	2.2
Community Health Worker	59	12.1
Duration since basic training completion		
Less than one year	12	4.5
One to four years	81	27.8
More than four years	181	67.7
Years of service at the facility		
Less than one year	41	16.5
One to four years	131	49.3
More than four years	102	34.2
Site		
Iganga Municipality	100	32.6
Jinja City	174	67.4

### Availability of family planning services

Of the 152 facilities included, 145 (94.2%, weighted proportion) reported offering at least one family planning service. Among these facilities, only 8.9% reported providing at least one permanent method, 34.2% provided long-acting reversible contraceptives (LARCs), and 36.1% offered LARC removal services. Additionally, 58.2% provided counselling on at least one natural family planning method, while nearly all (99.0%) offered at least one short-acting method (Table 3). Looking at specific methods, only 6.9% of facilities offered vasectomy, while 8.0% provided female sterilization. In contrast, most facilities supplied male condoms (95.5%) and combined oral contraceptive pills (85.4%). Injectable DMPA-IM was available in 76.5% of facilities, while self-injectable DMPA-SC was offered by 66.9%. Additionally, 21.3% provided IUD insertion, and 23.6% offered IUD removal services.

**Table 3:** Proportion of health facilities that reported providing the different methods by level of care (*N* = 145)

Methods	Weighted percentage of facilities						
	**Combined**	**Higher-level facility**	**HCIII**	**HCII**	**Pharmacy**	**Drug Shop**	**Clinic**
**Natural family planning method counselling**	**58.2**	**87.5**	**93.7**	**75.0**	**15.8**	**44.1**	**76.8**
Standard Days Method counselling	50.8	75.0	68.7	70.0	15.8	33.1	75.2
Lactational Amenorrhea Method counselling	52.5	75.0	93.7	65.0	15.8	35.4	73.6
Cycle Beads	16.0	25.0	50.0	20.0	6.9	18.6	28.5
**Short-acting methods**	**99.0**	**100.0**	**100.0**	**100.0**	**100.0**	**100.0**	**96.7**
Male condoms	95.5	100.0	87.5	95.0	100.0	98.1	91.7
Female condoms	24.6	37.5	25.0	45.0	6.9	22.4	26.7
Combined Oral Contraceptive Pills	85.4	100.0	87.5	80.0	79.1	85.8	86.6
Emergency Contraceptives	78.4	75.0	56.2	60.0	93.0	72.8	91.7
Injectable contraceptives—DMPA IM	76.5	100.0	81.2	80.0	52.5	67.2	93.5
Injectable contraceptives—DMPA SC	66.9	87.5	81.2	90.0	40.5	56.7	73.3
**Long-acting reversible contraceptive (LARC) methods**	**34.2**	**100.0**	**93.7**	**65.0**	**8.9**	**1.9**	**56.8**
IUD insertion	21.3	100.0	43.7	25.0	0.0	1.9	40.2
Contraceptive implant insertion	33.1	87.5	93.7	65.0	8.9	0.0	56.8
**LARC removal**	**36.1**	**100.0**	**93.7**	**65.0**	**8.9**	**1.6**	**63.5**
IUD removal	23.6	100.0	50.0	30.0	0.0	1.6	45.2
Contraceptive implant removal	36.1	100.0	93.7	65.0	8.9	1.6	63.5
**Permanent methods**	**8.9**	**75.0**	**12.5**	**5.0**	**0.0**	**0.0**	**16.8**
Female sterilization	8.0	75.0	0.0	5.0	0.0	0.0	16.8
Vasectomy	6.9	75.0	12.5	5.0	0.0	0.0	10.1

Comparing availability across different levels of care, higher-level facilities reported the highest availability for most family planning services. These included male condoms, oral contraceptive pills, DMPA-IM, UD insertion, and IUD and contraceptive implant removal which were offered in all higher-level facilities. HCIIIs also had high availability of male condoms (87.5%) and combined oral contraceptive pills (87.5%), though availability was lower for methods such as IUD insertion (43.7%) and female sterilization (0.0%). HCIIs also had high availability of male condoms (95.0%) and combined oral contraceptives (80.0%), but a lower availability of IUD insertion (25.0%) and female sterilization (5.0%).

Pharmacies and drug shops primarily stocked short-acting methods. Male condoms were available in all pharmacies and 98.1% of drug shops, while emergency contraceptives were available in 93.0% of pharmacies and 72.8% of drug shops. Injectable contraceptives—DMPA-IM and DMPA-SC—were available in 52.5% and 40.5% of pharmacies, respectively, and in 67.2% and 56.7% of drug shops. Notably, 8.9% of pharmacies and 1.9% of drug shops reported providing LARCs. Clinics exhibited moderate to high availability of most family planning methods, particularly short-acting methods such as male condoms and emergency contraceptives (91.7%) and DMPA-IM (93.5%). However, availability of long-term methods was lower compared to higher-level facilities. Specifically, IUD insertion was available in 40.2% of clinics, contraceptive implant insertion in 56.8%, and IUD removal in 45.2%. Permanent contraceptive methods were least available across all facility types (8.9%), with female sterilization provided in only 16.8% of clinics and vasectomy in 10.1%.

### Staff and guidelines

#### Staff training

Among the 145 facilities offering family planning services, 57.8% reported having at least one staff member providing family planning trained in some aspect of family planning within the past three years. Training levels varied by ownership type: only 50.4% of PFP facilities had a trained staff, compared to 75.0% of public facilities and 83.6% PNFP facilities.

From the health worker survey, 261 out of 274 respondents (95.3%) were from health facilities providing family planning services. However, out of the 261, few had received recent refresher training on family planning: 6.5% on tubal ligation, 7.1% on vasectomy, 20.2% on program management, and 26.3% on IUD procedures. Similarly, about a third had training in general clinical skills (37.8%), family planning counselling (39.5%), implant insertion and removal (33.8%), and LAM counselling (33.1%).

#### Knowledge of family planning methods

**Fig. 2:**
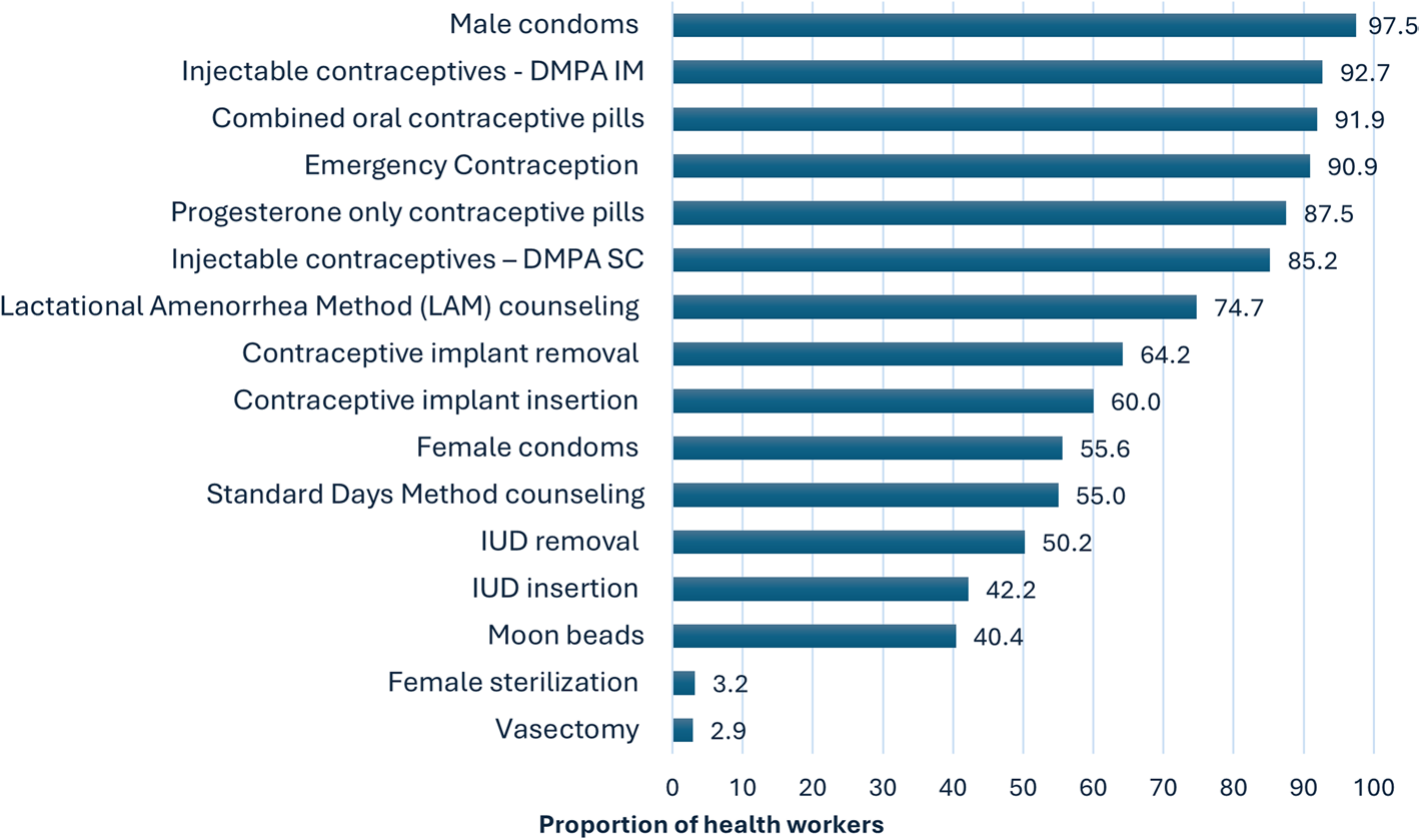
Proportion of health workers knowledgeable enough to counsel and provide various methods (*N* = 261)

Most health workers from the facilities that offered family planning (97.5%) were knowledgeable enough to counsel and provide male condoms (97.5%), DMPA-IM (92.7%), combined oral contraceptives (91.9%), and emergency contraceptives (90.9%) (Fig. 2). However, knowledge of vasectomy (2.9%), female sterilization (3.2%), IUD insertion/removal (42.4%/50.2%), natural methods SDM and moon beads, as well as that of the female condom, was limited, at 55.0%, 40.4%, and 55.6% respectively.

#### Guidelines

Only 10.1% of facilities offering family planning services had all relevant SRH and family planning guidelines, and 21.8% had family planning checklists. Guidelines were more available in PNFP (40.7%) and public (31.3%) facilities than in PFP facilities (1.2%). Checklists followed a similar pattern, with the highest availability in PNFP (68.3%) and public (59.4%) facilities compared to PFP facilities (21.8%).

### Equipment

Less than half (48.6%) of the facilities had a functioning blood pressure machine available on the day of data collection. The proportion was highest among clinics at 88.4%, followed by higher level facilities at 75.0%. HCIIs recorded a proportion of 55.0%, while drug shops had a proportion of 20.8%. Pharmacies had the lowest proportion of facilities with functioning blood pressure machines at 13.9%.

### Contraceptive supply and stock-out resilience

**Fig. 3:**
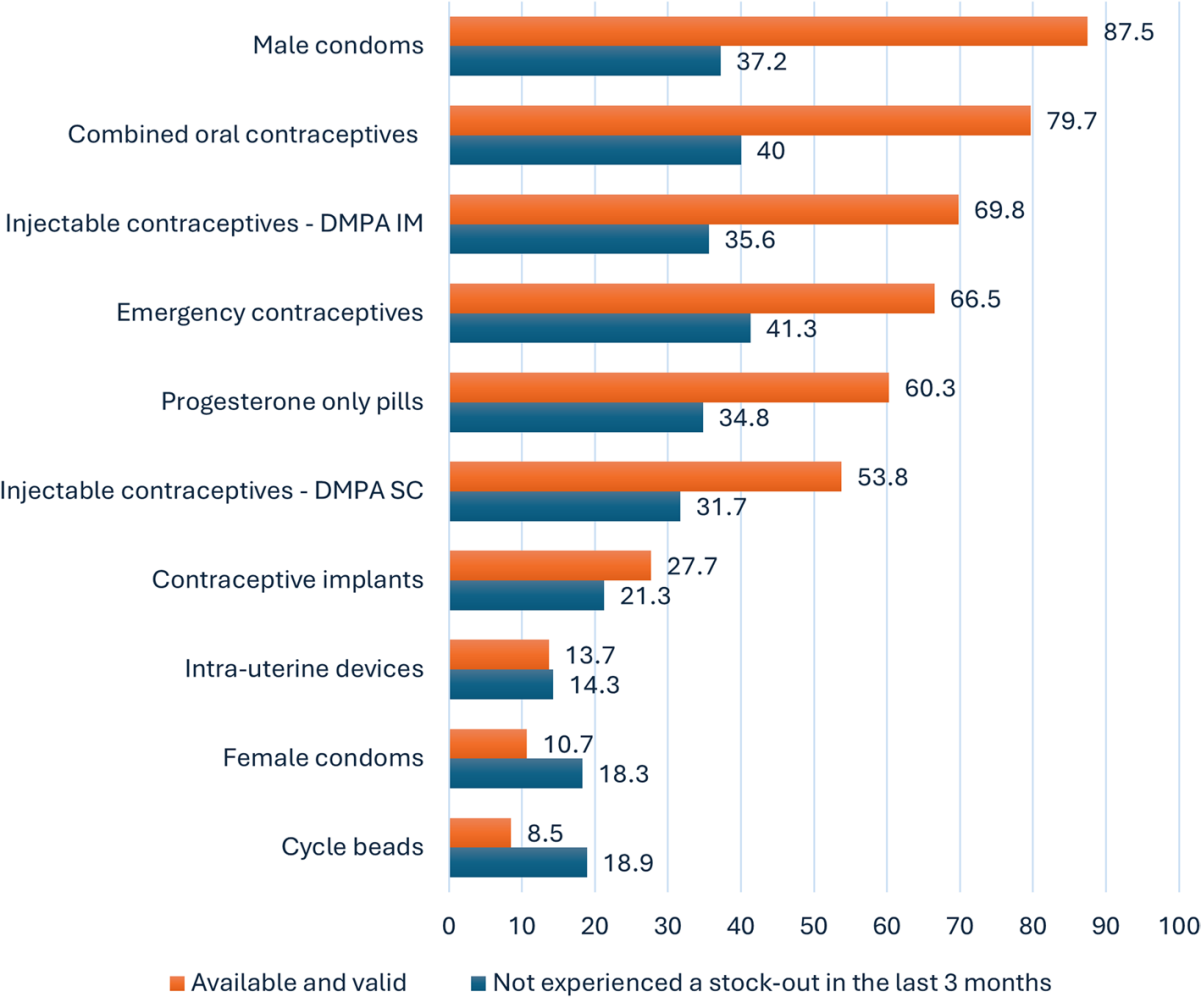
Proportions of facilities with different methods available and no stock-outs in the past three months (*N* = 145)

Most facilities assessed had male condoms (87.5%) and combined oral contraceptive pills (79.7%) available and valid (not expired or damaged) on the day of data collection (Fig. 3). However, only 37.2% of all facilities had avoided a male condom stock-out in the past three months, and 40.0% had avoided a combined oral contraceptive stock-out. Emergency contraceptives and injectable contraceptives, DMPA-IM also demonstrated relatively high availability, at 66.5% and 69.8%, respectively, but fewer than half of the facilities (41.3% for emergency contraceptives and 35.6% for DMPA-IM) reported no stock-outs. In contrast, IUDs, female condoms, and cycle beads had the lowest availability, at 13.7%, 10.7%, and 8.5%, respectively, with similarly low resilience to stock-outs (14.3%, 18.3%, and 18.9%, respectively).

### Governance of family planning services

Of the 145 facilities providing family planning services, only 24.4% had a designated focal person for SRH/family planning. Additionally, just 23.7% indicated that their family planning staff received external supervision to monitor performance, with 20.4% having received supportive supervision focused on SRH/family planning in the last three months. Furthermore, nearly half of the facilities (48.6%) did not hold formal meetings to discuss facility management.

### Service provision and imposed barriers to accessing family planning methods

**Fig. 4:**
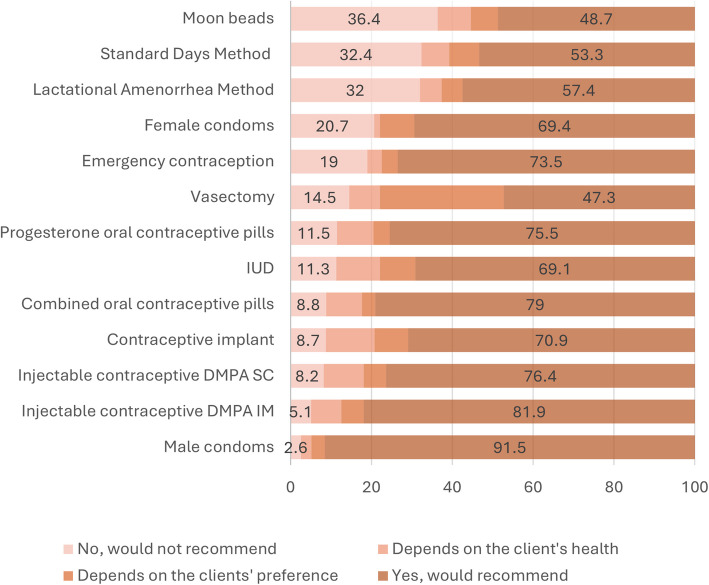
Weighted proportions of providers who reported recommending different family planning methods (*N* = 261)

Among the 261 health workers at family planning facilities, 89.9% reported providing family planning services in the past three months. When asked which methods they would never recommend under any circumstances, over a third indicated moon beads (36.4%), the SDM (32.4%), and the LAM (32.0%) (Fig. 4). Conversely, the most recommended methods were male condoms (91.5%), DMPA-IM (81.9%), DMPA-SC (76.4%), combined oral pills (79.0%), and progesterone-only pills (75.5%). Additionally, 30.6% would recommend vasectomy only if preferred by the client.

Among the providers, 97.7% imposed at least one restriction (minimum age, parity, marital status, or spousal consent) on clients seeking family planning methods. Minimum age requirements were set by 79.5% of providers, most commonly in PNFP facilities (89.6%), followed by public (80.5%) and PFP (73.9%) facilities (Table 4). Parity requirements were enforced by 59.5% of providers, with the highest at PNFP facilities (85.7%). Marital status restrictions were applied by 83.3% of providers, and 75.6% required spousal consent, with both restrictions most frequently imposed at PNFP facilities.

**Table 4:** Proportion of health workers imposing age, parity, marital status and spousal consent barriers and the minimum age and parity required (*N* = 261)

Access barrier						
**Method**	**Minimum age**		**Minimum parity**		**Marital status**	**Spousal consent**
	**Weighted proportion**	**Mean minimum age required ± SD**	**Weighted proportion**	**Mean number of children ± SD**	**Weighted proportion**	**Weighted proportion**
Any Method	79.5		59.5		83.3	75.6
Pills	48.7	17.0 ± 3.0	19.4	2.5 ± 1.4	63.9	23.6
Condom	36.3	16.2 ± 2.1	1.7	3.4 ± 1.2	48.4	29.2
IUD	50.7	18.2 ± 4.4	20.9	2.4 ± 2.0	65.0	39.2
Injectables	54.3	17.1 ± 3.2	25.8	1.8 ± 1.1	63.7	29.2
Sterilization	59.9	35.3 ± 10.1	48.2	6.1 ± 7.9	58.5	66.4

The proportion of health workers imposing restrictions was lowest for condom access and highest for sterilization, except regarding marital status. The mean minimum age requirement was lowest for condoms (16.2 years, SD ± 2.1) and highest for sterilization (35.3 years, SD ± 10.1). For minimum parity, injectables had the lowest requirement (1.8 children, SD ± 1.1), while sterilization required the highest (6.1 children, SD ± 7.9).

### Readiness to provide family planning services

**Fig. 5:**
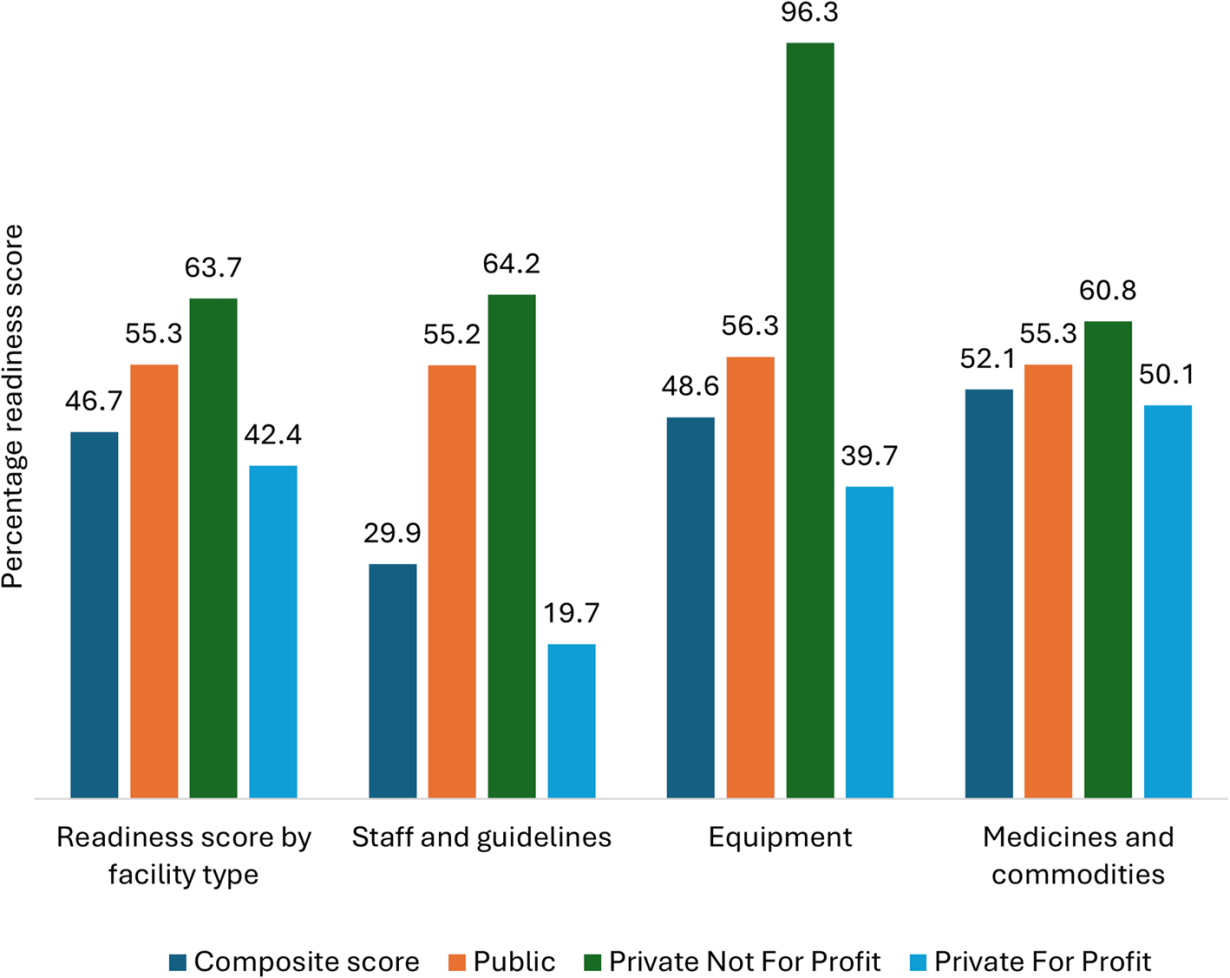
Readiness across public, PNFP and PFP facilities in Jinja City and Iganga Municipality

The average readiness score was 46.7% (SD ± 17.0) (Fig. 5). The lowest mean scores were in the staff and guidelines domain (29.9%, SD ± 29.1%), followed by equipment (48.6%, SD ± 50.1), while the highest scores were in medicines and commodities (52.1%, SD ± 18.2). PFP facilities had the lowest mean composite score across all dimensions at 42.4% (SD ± 14.1). Public health facilities followed with a mean score of 55.3% (SD ± 20.1), and PNFP facilities had the highest mean score at 63.7% (SD ± 15.3).

### Factors associated with readiness

In the bivariate analysis, factors such as the study site, facility ownership, level of care, receipt of outside supervision to monitor performance of family planning services, holding administrative meetings, and the proportions of health workers imposing marital status and spousal consent barriers to service access at the facilities were all associated with readiness to provide family planning services (Table 5).

**Table 5:** Factors associated with readiness to provide family planning services (*N* = 144)

Variable	Unadjusted coefficient	Confidence interval	*p-*value	Adjusted coefficient	Confidence interval	*p-*value
Site						
Iganga Municipality	Reference				Reference	
Jinja City	9.53	4.00 to 15.06	< 0.001	4.42	−0.42 to 9.25	0.073
Ownership						
Public	Reference					
Private-Not-For-Profit	7.73	−2.29 to 17.75	0.130			
Private-For-Profit	−12.86	−19.28 to −6.45	< 0.001			
Level of facility						
Higher level facility	Reference				Reference	
Health Centre III	−4.69	−15.93 to 6.55	0.411	1.53	−9.48 to 12.54	0.784
Health Centre II	−16.01	−24.73 to −7.29	< 0.001	−9.42	−18.24 to −0.06	**0.036**
Private pharmacy	−22.14	−30.99 to −13.29	< 0.001	−4.44	−15.19 to 6.31	0.415
Private drug Shop	−27.49	−33.95 to −21.03	< 0.001	−11.00	−20.36 to −1.63	**0.022**
Private clinic	−13.37	−21.69 to −5.04	0.002	−0.73	−9.46 to 8.01	0.870
Slum location						
No	Reference					
Yes	−5.06	−11.88 to 1.76	0.144			
Staff receive outside supervision						
No	Reference				Reference	
Yes	15.81	9.17 to 22.47	< 0.001	9.04	2.31 to 15.77	**0.009**
Administrative meetings						
None	Reference				Reference	
Meetings held	19.98	11.36 to 28.60	< 0.001	9.72	1.64 to 17.80	**0.019**
Proportion imposing a minimum age barrier	1.04	−5.09 to 7.18	0.737			
Proportion imposing a minimum parity barrier	3.79	−2.44 to 10.02	0.231			
Proportion imposing a marital status barrier	−12.04	−22.91 to −1.17	0.030	−9.42	−17.15 to −1.69	**0.017**
Proportion imposing a spousal consent	8.28	2.07 to 14.49	0.009	6.24	0.88 to 11.61	**0.023**

However, after adjusting for other variables, only the level of facility, receipt of outside supervision for family planning services, holding administrative meetings, and an increase in the proportions of health workers imposing marital status and spousal consent barriers to service access remained significantly associated with readiness. Compared to higher-level facilities, HCII facilities and drug shops had significantly lower readiness to provide family planning services (β = −9.42, *p* = 0.036) and (β = −11.00, *p* = 0.022), respectively. HCIIIs, pharmacies and clinics did not differ significantly from the higher-level facilities. Receiving external supervision for family planning services was associated with a significant increase in readiness (β = 9.04, *p* = 0.009). Similarly, holding regular administrative meetings was positively associated with readiness (β = 9.72, *p* = 0.017). Regarding provider-level barriers, increase in the proportion of providers imposing marital status barriers was negatively associated with readiness (β = −9.42, *p* = 0.017), while imposing spousal consent requirements was positively associated (β = 6.24, *p* = 0.023).

## Discussion

This study aimed to assess the readiness of health facilities to provide family planning services in urban east-central Uganda. Despite a high availability of services, the findings revealed a low overall readiness, especially in the staff and guidelines domain, with the lowest scores observed in PFP facilities. Short-term contraceptive methods were relatively widely available, but the availability of long-term and permanent methods at the assessed facilities was significantly lower, compounded by frequent stock-outs. Furthermore, the study found that refresher training for staff was insufficient, particularly in PFP facilities. This inadequacy was reflected in low levels of knowledge, confidence and willingness to provide various family planning methods, especially long-term options and counselling on natural family planning. Additionally, there were prevalent biases against certain methods and restrictions on access based on age, parity, marital status, and spousal consent, especially in PNFP facilities. Importantly, the analysis indicated that readiness was significantly associated with the facility level, receipt of outside supervision for family planning services, holding administrative meetings, and imposing marital status and spousal consent barriers.

The 94.2% availability of family planning services found in this study aligns with findings by Ali, Farron [9], who reported 96% availability in urban Ugandan health facilities, and the 2023 Uganda Harmonized Health Facility Assessment report, which found 95% in the Busoga region [36]. These figures reflect the success of interventions by the Ministry of Health and partners to improve access. However, readiness scores were low, consistent with findings from Uganda [36] and other African countries [8, 9]. HCII facilities and drug shops, particularly, scored significantly lower than higher-level facilities. This is concerning as HCIIs make up the largest proportion of the public health infrastructure and are the closest source of family planning for many. Likewise, private drug shops are widespread in Uganda’s urban areas, where access to public health services is limited, primarily due to their lower start-up costs and minimal regulatory requirements. While the Ministry of Health’s Strategic Plan 2020/21—2024/25 aims to enhance HCII functionality and preparedness through upgrades [37], our findings still highlight the need for a balanced approach to increasing access to family planning. Efforts to expand availability through diverse providers, as outlined in the TMA [22] must be matched by an equal commitment to ensuring the readiness of all facilities, especially lower-level ones, to effectively deliver services.

From a rights-based perspective, it is important for facilities to provide a diverse method mix to support individual choice and autonomy [15]. However, this study found that while short-acting methods were relatively available, long-acting and permanent methods were not, a finding consistent with the Harmonized Health Facility Assessment [36]. This study also found a high prevalence of commodity stock-outs, consistent with reports from Uganda [12] and other countries [9]. These stock-outs limit the contraceptive method mix, restricting client choice and create opportunities for provider coercion. The scarcity of long-acting methods, despite their high effectiveness and convenience [38], is particularly concerning as it heightens the risk of unintended pregnancies. Their availability was especially low in private clinics (56.8%), where service provision is often driven by demand, profitability and financial resources. Notably, some HCII facilities, private drug shops, and pharmacies offered long-acting methods despite policies restricting them from doing so [28, 29]. Given the low readiness levels in these settings, regular supervision is essential to ensure compliance with regulations and safeguard clients accessing services from such facilities.

This study found that the proportion of staff who had received refresher training on family planning was low, particularly in PFP facilities. This gap in training likely accounts for the observed low levels of knowledge and confidence and willingness to provide various methods, especially the long-acting options and counselling on natural family planning. Similar findings of low provider knowledge about family planning, linked to a lack of refresher training, have been reported in other parts of Uganda [39]. These results highlight an urgent need for targeted capacity-building efforts, including refresher sessions and on-job mentorship, to reinforce competency to provide the full range of services. And, while the Ministry of Health may prioritize training in public facilities, private providers, who make up a significant share of urban service providers [16], should also be included in capacity-building efforts, especially in light of new methods like DMPA-SC.

We found that readiness was influenced by governance-related factors at health facilities, such as receiving external supervision and conducting management meetings, both of which were associated with increased readiness. Similarly, Rahman, Islam [8] observed that regular facility management meetings and external supervision visits were linked to higher readiness scores in other African countries. These findings highlight the importance of effective governance of family planning services, both within facilities and by oversight bodies such as city and municipal health authorities, in improving service readiness. However, Uganda’s private health sector faces significant governance challenges, including inadequate regulatory inspections in drug shops, bribery during inspections, and insufficient resources for supervisors to perform their duties effectively [17]. In this context, regular external supervision becomes particularly relevant. It not only helps enforce standards but also addresses the private sector’s reluctance to invest in essential items, such as blood pressure machines and guidelines, which are vital for quality family planning services but may be deemed non-essential in settings like drug shops. Moreover, supervision can help address skills gaps, especially when refresher training opportunities are limited.

Our findings revealed provider biases against specific family planning methods, with the highest against natural methods like SDM, LAM, and moon beads, and female condoms. These biases aligned with the low knowledge levels about these methods, suggesting that inadequate knowledge may fuel negative attitudes. However, beyond knowledge gaps, provider biases may also be shaped by clinical experience, where past observations of side effects or method discontinuation influence how providers counsel clients. While such experiences are valuable, they can lead to overly cautious recommendations or selective counselling, ultimately restricting client autonomy. Similar findings from rural Ethiopia indicated that providers sometimes steered women towards long-acting methods by emphasizing perceived disadvantages of short-acting options or sharing inaccurate information [40]. Although provider judgment plays a crucial role in matching clients with suitable methods, it must be guided by evidence-based counselling that presents all options impartially, empowering clients to make informed choices.

Our study also uncovered provider-imposed barriers to family planning access based on minimum age, parity, marital status, and spousal consent, particularly in PNFP facilities. The proportion of providers imposing these barriers was notably higher than reported in urban Kenya [41] and Burkina Faso [42], likely due to differences in socio-cultural contexts. In PNFP facilities, such restrictions may indicate an organizational culture influenced by, especially, religious affiliations as many are faith-based, fostering a discriminatory environment despite the facilities’ higher readiness to provide services. At an individual level, these actions or beliefs may stem from social or religious convictions, misinformation about contraceptive safety, or paternalistic attitudes, where providers act in what they perceive as the clients’ best interests [43, 44]. These biases also suggest that service providers reinforce patriarchal gender norms, viewing male spouses as primary decision-makers in their partners’ reproductive health choices, a dynamic that has been linked to lower contraceptive use [45, 46]. Moreover, while national policies broadly endorse family planning access for adolescents [28, 47], the absence of explicit operational guidance, particularly on consent, confidentiality, and eligibility for minors, leaves room for restrictive interpretations. These findings highlight the need to address both structural readiness and health worker attitudes to ensure equitable access for adolescents, non-parous women, unmarried women, and those using family planning covertly. Addressing these barriers requires formulating and enforcing clear national guidelines that prohibit discriminatory practices coupled with training for public and private sector health workers to address personal biases and promote adherence to non-discriminatory policies.

### Study strengths and limitations

This study is one of the few to assess readiness across both public and private facilities, capturing the full spectrum of providers in Uganda, which enhances the generalizability of the findings. By assessing both health facilities and workers, the study also provides a holistic analysis that considers both institutional and individual-level factors influencing service delivery.

However, there are some limitations to consider. The use of secondary data may have introduced biases inherent in the original study’s design, particularly regarding the representativeness of the included health facilities and workers. To mitigate this, we applied sampling weights to enhance representativeness of our findings. Second, the limited sample size may have led to imprecise estimates, as reflected in the wide confidence intervals for some variables, potentially affecting the detection of associations. However, the sample size was calculated appropriately based on the total number of eligible health facilities in the study area. Future studies could consider a broader geographical scope to include more facilities and improve the precision and generalizability of findings. Third, reliance on self-reported data from health workers about their biases and knowledge could be subject to social desirability bias, potentially inflating estimates of their readiness. To minimise this, we relied on the SARA methodology as an objective measure of readiness and contextualized self-reported data within broader facility-level findings. Finally, as a secondary analysis, this study was constrained by the dataset’s variables, limiting exploration of additional factors like supply chain dynamics beyond the facility and socio-cultural influences. Despite this, triangulating multiple data sources strengthened the rigor of our assessment.

## Conclusions

While the availability of family planning services in urban east-central Uganda was found to be high, the findings highlight significant gaps in facility readiness, particularly in staff capacity, commodity availability and the provision of long-term contraceptive methods. These gaps are especially pronounced in lower-level facilities (HCIIs and private drug shops), which are vital sources of family planning services in urban areas.

Addressing these challenges requires an integrated approach. First, stronger accountability mechanisms, especially for private facilities, are needed to ensure compliance with standards. Second, increased investment in the capacity building of mainly lower-level facilities, particularly HCIIs and private drug shops, is crucial to improving service delivery. This should include targeted training programs for both public and private sector providers to enhance their ability to provide a full range of contraceptive options. Finally, policies should prioritise ensuring that facilities are equipped to deliver high-quality, comprehensive family planning services within their scope, free from discrimination. Importantly, expanding service access cannot come at the expense of quality; urban family planning success hinges on simultaneously improving both service availability and facility readiness.

## Supplementary Information

Supplementary Material 1.

## Data Availability

Data analysed for this study are available on request. Send data request to sphrecadmin@musph.ac.ug.

## Abbreviations

CHW: Community Health Worker
DMPA: Depot Medroxyprogesterone Acetate
HC: Health Centre
IM: Intramuscular
IUD: Intrauterine Device
LAM: Lactational Amenorrhea Method
PFP: Private-for-Profit
PNFP: Private-not-for-Profit
SC: Subcutaneous
SDM: Standard Days Method
