# Supplementary material for: Readiness of health facilities to deliver family planning services and associated factors in urban east-central Uganda
Source: Reprod Health. 2025 May 15;22:82. doi: 10.1186/s12978-025-02026-w (PMC12083106; doi:10.1186/s12978-025-02026-w)
Supplement: Supplementary file 1 — Supplementary Material 1. [file 12978_2025_2026_MOESM1_ESM.docx]

**Additional File 1: Tracer indicators used to generate the readiness scores**

| **Domain** | **Tracer Indicator** | **Criteria** | **Code** | **Reference Question** | **Yes** | **No** | **Max. Total** |
| --- | --- | --- | --- | --- | --- | --- | --- |
| Staff and guidelines | Guidelines on family planning | Guidelines observed in service area | SG1 |  | 1 | 0 | 1 |
|  | Family planning checklists and/or job aids | Availability of the family planning screening checklist (WHO or Ministry of Health contraceptive wheel) | SG2 | L7. Go to the room where FP counselling takes place. Are the following job aids available? FP Screening Checklists (WHO or Ministry of Health contraceptive wheel) | 1 | 0 | 1 |
|  | Staff trained in family planning | At least one staff member providing the service trained in the last two years in some aspect of family planning | SG3 | I3. I would like to know if the following basic equipment items were available and function in the last quarter and today. *(Ask to see the items and observe if it is functioning)*  Blood pressure apparatus (may be digital or manual sphygmomanometer with stethoscope | 1 | 0 | 1 |
| Equipment | Blood pressure apparatus | Digital BP machine or manual sphygmomanometer with stethoscope observed availability, reported functionality, and in service area or adjacent area. | EQ1 | P1. I would like to know if the following basic equipment items were available and function in the last quarter and today. *(Ask to see the items and observe if it is functioning begin with AVAILABLE AND FUNCTIONAL TODAY?)* Blood pressure apparatus (may be digital or manual sphygmomanometer with stethoscope) | 1 | 0 | 1 |
| Medicines and commodities | Combined estrogen progesterone oral contraceptive pills | Observed in the service area or where routinely stored in stock with at least one valid. | MC1 | N1. Of the contraceptive methods provided at this facility, which are available today?  If the method is available, check to see if it is VALID (Not expired or damaged).  Oral contraceptives-COCs | 1 | 0 | 1 |
|  | Progestin-only contraceptive pills | Observed in service area OR where routinely stored; in stock with at least one valid. | MC2 | N1. Of the contraceptive methods provided at this facility, which are available today?  If the method is available, check to see if it is VALID (Not expired or damaged).  Oral contraceptives-POPs | 1 | 0 | 1 |
|  | Injectable contraceptives (DMPA-IM) | Progestin-only injectable contraceptive,  DMPA-IM, available in stock with at least 1 valid. | MC3 | N1. Of the contraceptive methods provided at this facility, which are available today?  If the method is available, check to see if it is VALID (Not expired or damaged).  Injectable contraceptives – DMPA IM | 1 | 0 | 1 |
|  | Condoms (Male) | Observed in service area OR where routinely stored; in stock with at least one valid. | MC4 | N1. Of the contraceptive methods provided at this facility, which are available today?  If the method is available, check to see if it is VALID (Not expired or damaged).  Male condoms | 1 | 0 | 1 |
| Other family planning commodities in stock | Condoms (Female) | Observed in service area OR where routinely stored; in stock with at least one valid. | MC5 | N1. Of the contraceptive methods provided at this facility, which are available today?  If the method is available, check to see if it is VALID (Not expired or damaged).  Female condoms | 1 | 0 | 1 |
|  | Progestin-only injectable contraceptives (DMPA-SC) | Observed in service area OR where routinely stored; in stock with at least one valid. | MC6 | N1. Of the contraceptive methods provided at this facility, which are available today?  If the method is available, check to see if it is VALID (Not expired or damaged).  Injectable contraceptives – DMPA-SC | 1 | 0 | 1 |
|  | Implants^c^ | Observed in service area OR where routinely stored; in stock with at least one valid. | MC7 | N1. Of the contraceptive methods provided at this facility, which are available today?  If the method is available, check to see if it is VALID (Not expired or damaged).  Contraceptive implants | 1 | 0 | 1 |
|  | Emergency contraceptives | Observed in service area OR where routinely stored; in stock with at least one valid. | MC8 | N1. Of the contraceptive methods provided at this facility, which are available today?  If the method is available, check to see if it is VALID (Not expired or damaged).  Emergency contraception | 1 | 0 | 1 |
|  | Intra-uterine contraceptive devices (IUCDs)^d^ | Observed in service area OR where routinely stored; in stock with at least one valid. | MC9 | N1. Of the contraceptive methods provided at this facility, which are available today?  If the method is available, check to see if it is VALID (Not expired or damaged).  IUDs | 1 | 0 | 1 |
| Stock-outs in the past 3 months | Female condoms |  | SO1 | N2. Of the contraceptive methods provided at this facility, which have experienced a stockout in the last 3 months?  *To determine if there has been a stockout in the last 3 months, you may (1) ask a staff member in charge of FP services or the person in charge of logistics, and/or (2) verify if any method has been out of stock by checking records, if available. If a stockout is indicated, either by a staff member or by the records, choose ‘yes’ even if the method is available today. If the method is not offered select "NA".*  Female Condoms | 0 | 1 | 1 |
|  | Implants^e^ |  | SO2 | N2. Of the contraceptive methods provided at this facility, which have experienced a stockout in the last 3 months?  Contraceptive implants | 0 | 1 | 1 |
|  | Emergency contraceptives |  | SO3 | N2. Of the contraceptive methods provided at this facility, which have experienced a stockout in the last 3 months?  Emergency contraception | 0 | 1 | 1 |
|  |  |  |  | **Total Score:**  Hospitals, HCIV, HCIII and clinics = (n/16)*100  Pharmacies, drug shops and HCIIs = (n/13)*100 | | | |
| **Notes:**  ^c^MC7: Not assessed for pharmacies, drug shops and HCII  ^d^MC9: Not assessed for pharmacies, drug shops and HCII  ^e^SO2: Not assessed for pharmacies, drug shops and HCII  Combined estrogen-progesterone injectable contraceptives was removed because it is not routinely provided in health facilities in Uganda. | | | | | | | |
